# Change Fatigue and Attitudes Toward AI in County‐Level Nurses: The Mediating Role of AI Literacy

**DOI:** 10.1155/jonm/4386800

**Published:** 2026-06-30

**Authors:** Ming Yu, Mengjia Zhou, Rong Yu, Xiaoli Fan, Ronghui Geng, Jing Ji, Suping Cai, Lili Jiang, Lingling Jiang

**Affiliations:** ^1^ Department of Nursing, Affiliated Rudong Hospital of Xinglin College, Nantong University, Nantong, Jiangsu, 226001, China, ntu.edu.cn

**Keywords:** AI application attitudes, artificial intelligence literacy, change fatigue, county-level nurses, mediating effect, nursing management

## Abstract

**Objective:**

Grounded in the conservation of resources theory, this study aimed to examine the mediating role of artificial intelligence literacy in the relationship between change fatigue and attitudes toward AI application among nurses in county‐level hospitals, thereby providing insights for enhancing AI technology acceptance in primary care settings.

**Background:**

County‐level hospitals in China are undergoing significant organizational transformations alongside rapid technological advancements. Nurses in these settings frequently experience change fatigue due to continuous institutional reforms, while simultaneously facing challenges in adapting to artificial intelligence technologies. Understanding the psychological mechanisms underlying nurses’ acceptance of AI is crucial for successful technology implementation in primary care settings.

**Design:**

A cross‐sectional analytic study employing mediation analysis.

**Methods:**

A cross‐sectional survey was conducted using convenience sampling from August to September 2025. A total of 460 clinical nurses from a county‐level tertiary B hospital in Nantong City, China (99.6% female; mean age 31–40 years: 43.3%), were assessed. Data were analyzed using structural equation modeling with the maximum likelihood estimation method, and the significance of indirect effects was tested using the bootstrap method (5000 samples).

**Results:**

The mean scores for change fatigue, AI literacy, and AI application attitudes were 29.27 ± 6.98, 49.82 ± 5.27, and 45.26 ± 2.45, respectively. Change fatigue showed a significant negative association with AI application attitudes (*β* = −0.36, *p* < 0.001), while AI literacy demonstrated a significant positive effect (*β* = 0.34, *p* < 0.001). AI literacy partially mediated the relationship between change fatigue and AI application attitudes (indirect effect = −0.185, 95% CI: [−0.231, −0.142]), accounting for 37.7% of the total effect.

**Conclusion:**

AI literacy plays a significant partial mediating role. To improve nurses’ acceptance of AI technologies, nursing administrators should implement dual interventions aimed at both alleviating change fatigue (e.g., paced change management and psychological support) and systematically enhancing AI literacy (e.g., stratified training programs).

## 1. Introduction

The rapid integration of artificial intelligence (AI) into healthcare has positioned nurses as critical adopters whose attitudes directly influence the success of AI implementation [1]. Substantial research has examined nurses’ AI attitudes, acceptance, and readiness across clinical domains such as critical care and chronic disease management [2, 3]. These studies have also identified the importance of AI literacy and training [4], as well as barriers including ethical concerns and workflow disruptions [5, 6]. Evidence further shows that ethical awareness moderates the relationship between nurses’ AI perceptions, their attitudes, and innovative behavior [7]. However, most existing research has focused on individual‐level factors. It has largely overlooked the broader organizational context.

Many healthcare systems in China, especially primary care services, undergo constant organizational reforms including policy adjustments and system improvements. This environment can precipitate change fatigue, a state of psychological resource depletion resulting from sustained adaptation efforts, which is linked to burnout and diminished learning motivation [8, 9]. Grounded in the conservation of resources (COR) theory [10], individuals strive to protect finite psychological resources. We posit that chronic resource drain from change fatigue may impair two resource‐intensive processes. Both processes are essential for AI integration. The first is the cognitive capacity to acquire AI literacy. The second is the emotional reserve to maintain an open attitude toward yet another workplace change—the introduction of AI.

International comparison highlights the uniqueness of China’s county‐level hospitals. Unlike primary care settings in Western countries, which often benefit from more gradual technology rollouts and better staffing support [11], county‐level hospitals in China undergo rapid, simultaneous reforms (e.g., hospital mergers and electronic record overhauls) with severe resource constraints [12]. Thus, nurses in this context face compounded pressures: high change fatigue and limited opportunities to develop AI literacy. This makes them a particularly vulnerable yet strategically important population for studying the mechanisms linking organizational change to technology acceptance.

Although previous studies have separately confirmed the role of AI literacy in technology acceptance [4, 13] and the detrimental effects of organizational change on employee well‐being [8, 14], a significant gap remains: how does generalized change fatigue, as a contextual antecedent, erode nurses’ AI literacy and thereby shape their attitudes toward AI? This pathway is especially salient in county‐level hospitals yet has received little empirical attention [12].

We developed and tested a mediation model with change fatigue as the independent variable, AI application attitude as the dependent variable, and AI literacy as the mediator. The study aims to provide theoretical insights grounded in the COR theory and practical guidance for AI implementation in resource‐limited primary care settings.

## 2. Methods

### 2.1. Study Design

This descriptive, cross‐sectional study tested a simple mediation model using quantitative data from validated self‐report scales, in which AI literacy was hypothesized to mediate the relationship between change fatigue and attitudes toward AI application. This study was reported in accordance with the STROBE (Strengthening the Reporting of Observational Studies in Epidemiology) checklist for cross‐sectional studies.

### 2.2. Participants and Settings

This cross‐sectional study was conducted in a county‐level tertiary B hospital in Nantong City, Jiangsu Province, China. County‐level hospitals serve as the critical hubs of China’s primary healthcare system, making them strategic settings for investigating the attitudes of nurses who are at the frontline of grassroots medical services. A convenience sampling method was employed to recruit clinical nurses between August and September 2025.

Inclusion criteria were (1) being a registered clinical nurse; (2) having at least 6 months of clinical nursing experience; (3) providing informed consent. Exclusion criteria were (1) nurses undergoing further training or internships; (2) nonclinical staff (e.g., nursing department administrators).

The sample size was calculated using the formula *n* = [(Zα/2 × CV)/*ε*]^2^, where Zα/2 = 1.96, CV = SD/mean, and *ε* = 2%. Based on a previous study by Hu et al. [15], CV was estimated as 0.1556. Accounting for a 20% invalid response rate, the minimum required sample size was 274.

Furthermore, for structural equation modeling (SEM), it is recommended to have a sample size of at least 10–20 cases per estimated parameter. Our hypothesized model included 23 estimated parameters, suggesting a minimum sample of 230–460 cases. Therefore, our final sample of 460 nurses was considered more than adequate for achieving sufficient statistical power to detect the hypothesized effects.

### 2.3. General Information Questionnaire

A self‐developed general information questionnaire was used to collect demographic and professional characteristics of the clinical nurses. The collected data included age, gender, educational level, professional title, frequency of monthly night shifts, and whether they had participated in AI‐related training. The selection of these items was based on a comprehensive literature review of studies on nurses’ attitudes toward technology and change fatigue [8, 16], as well as expert consultation, following established instrument development methodology. Content validity was assessed by a panel of three senior nursing managers and two nursing researchers, who evaluated the relevance and clarity of each item. The item‐level content validity index (I‐CVI) ranged from 0.80 to 1.00, and the scale‐level content validity index (S‐CVI/Ave) was 0.93, indicating good content validity. This questionnaire was pilot‐tested on 30 clinical nurses (not included in the main study sample) to ensure clarity and comprehensibility, and no modifications were required based on their feedback. Content validity was assessed using the CVI method, with I‐CVI ≥ 0.78 and S‐CVI/Ave ≥ 0.90 considered acceptable [17, 18].

### 2.4. Attitude Scale Towards the Use of AI Technologies in Nursing (ASUAITIN)

ASUAITIN, originally developed by Dilek Yılmaz et al. [19], was used to assess nurses′ attitudes toward AI technology application. The Chinese version of the scale was cross‐culturally adapted and validated by Hu et al. in a sample of 499 clinical nurses in Sichuan Province, demonstrating good psychometric properties [15]. The scale comprises 15 items distributed across two subscales: positive attitude (9 items) and negative attitude (6 items). The positive attitude subscale reflects a tendency to recognize and accept the benefits, value, and prospects of AI in nursing, whereas the negative attitude subscale captures concerns and unease regarding potential risks, ethical issues, and professional impacts associated with AI applications. All items are rated on a 5‐point Likert scale ranging from 1 (strongly disagree) to 5 (strongly agree). The total score ranges from 15 to 75, with a theoretical midpoint of 45; higher scores indicate more positive attitudes toward the application of AI technologies. In the original validation study, the overall Cronbach’s *α* was 0.910. In the present study, the Cronbach’s *α* for the total scale was 0.845.

### 2.5. AI Literacy Scale (AILS)

AILS, developed by Wang et al., was used to assess AI literacy in the general population [4]. This study used the Chinese‐translated version of the AILS, which was cross‐culturally adapted and validated among Chinese clinical nurses by Kong et al. [20]. The scale consists of 12 items distributed across four dimensions: Awareness, Use, Evaluation, and Ethics, with each dimension containing 3 items. Responses are recorded on a 7‐point Likert scale ranging from 1 (strongly disagree) to 7 (strongly agree). The total score ranges from 12 to 84, with higher scores indicating a higher level of AI literacy. The scale demonstrated good internal consistency with a Cronbach’s *α* coefficient of 0.821. In the present study, the Cronbach’s *α* coefficient for the AILS was 0.858.

### 2.6. The Six‐Item Change Fatigue Measurement Scale

The Six‐item Change Fatigue Measurement Scale, originally developed by Beernerth et al. and cross‐culturally adapted into Chinese by Zhang Xinyue et al., was used to measure change fatigue levels among clinical nurses who had experienced organizational changes in hospital environments [21]. The scale consists of 6 items, each rated on a 7‐point Likert scale ranging from 1 (strongly disagree) to 7 (strongly agree). The total score ranges from 6 to 42, with higher scores indicating more severe change fatigue. In the original validation study, the scale demonstrated good internal consistency with a Cronbach’s *α* coefficient of 0.85. In the present study, the Cronbach’s *α* coefficient for this scale was 0.910.

### 2.7. Data Collection

Data were collected via electronic questionnaires distributed through WeChat. The preface outlined the study purpose, instructions, and privacy protection. Participation was voluntary, and informed consent was obtained. An anonymous survey mechanism restricted each user to a single submission. Questionnaires with a completion time of less than 120 s or showing patterned responses were considered invalid and excluded. A total of 478 questionnaires were returned, yielding 460 valid responses (effective response rate: 96.23%).

### 2.8. Statistical Analysis

Data analysis was performed using SPSS 26.0 and Amos 26.0. Descriptive statistics were computed for all variables. The Harman single‐factor test was initially employed to examine common method bias. Furthermore, given the extremely high inverse correlation between the positive and negative attitude subscales (*r* = −0.916), which suggests they represent opposite poles of a single underlying dimension, the total score of the ASUAITIN scale was used in all subsequent mediation analyses to avoid multicollinearity and to align with a unidimensional interpretation of the attitude construct. Pearson correlation analysis was used to assess bivariate relationships. SEM with the maximum likelihood estimation method was applied for mediation analysis, with the Bootstrap method (5000 samples) utilized to test the significance of the indirect effects. To more rigorously assess common method bias, the unmeasured latent method factor approach was additionally employed within the SEM framework. The model fit was evaluated using indices including *χ* ^2^/df < 3.000, RMSEA < 0.080, and AGFI, NFI, CFI, IFI > 0.900.

In preliminary analyses, we examined models that incorporated key demographic and professional variables (e.g., age, education, income, night shift frequency, AI training) as covariates. The inclusion of these covariates did not significantly improve model fit indices, and the primary path coefficients and their significance levels remained largely unchanged. Therefore, to maintain model parsimony and clarity of presentation, the final mediation model is reported without covariates.

### 2.9. Minimization of Bias

To minimize potential researcher bias, several strategies were employed. First, all data were collected anonymously using self‐administered questionnaires, which reduces social desirability bias. Second, the research team members involved in data collection were not involved in the direct clinical supervision of the participants, minimizing the risk of coercion or response distortion. Third, the Harman single‐factor test and the unmeasured latent method factor approach were used to assess and control for common method bias (see Section 2.6). Fourth, the statistical analysis was conducted independently by two researchers (Ming Yu and Jing Ji), and any discrepancies were resolved through discussion with a third researcher (Lingling Jiang) to ensure accuracy and objectivity.

## 3. Results

### 3.1. General Information of Clinical Nurses

The demographic and professional characteristics of the participants are summarized in Table 1. The sample consisted predominantly of female nurses (99.6%).

**TABLE 1:** General information of the survey subjects (*n* = 460).

Project		*N (%)*
Gender	Male	2 (0.4)
	Female	458 (99.6)

Age (years)	≤ 25	70 (15.2)
	26–30	126 (27.4)
	31–40	199 (43.3)
	> 40	65 (14.1)

Educational background	Secondary specialized school	139 (30.2)
	Junior college	234 (50.9)
	Bachelor’s degree or higher	87 (18.9)

Length of service (years)	≤ 3	137 (29.8)
	4∼6	82 (17.8)
	7∼10	70 (15.2)
	11∼15	69 (15)
	≥ 15	102 (22.2)

Professional title	Junior professional title and below	253 (55)
	Intermediate professional title and below	192 (41.7)
	Senior professional titles and below	15 (3.3)

Monthly income (RMB)	≤ 4000	79 (17.2)
	4001∼6000	150 (32.6)
	6001∼8000	72 (15.7)
	≥ 8000	159 (34.6)

Number of night shifts per month	0	105 (22.8)
	1∼3	44 (9.6)
	4∼9	291 (63.3)
	≥ 10	20 (4.3)

Participate in AI‐related knowledge training	Have	58 (12.6)
	None	402 (87.4)

### 3.2. AI Application Attitude, Change Fatigue, and AI Literacy Scores

Table 2 presents the descriptive statistics for all key study variables.

**TABLE 2:** Scores of the clinical nurse change fatigue scale, AILS scale, and AI application attitude scale ($\overset{¯}{X}\pm S$, *n* = 460).

Project	Minimum	Maximum	Total score	Skewness	Kurtosis
Change fatigue	12	39	29.27 ± 6.98	−1.21	0.457
Attitude toward AI applications	38	51	45.26 ± 2.45	0.014	−0.434
Positive attitude	10	38	23.07 ± 5.54	0.422	−0.55
Negative attitude	10	32	22.19 ± 4.04	−0.08	−0.62
AI literacy	35	65	49.82 ± 5.27	0.276	−0.503
Consciousness	9	17	12.68 ± 1.95	0.018	−0.564
Using	9	16	12.26 ± 1.26	−0.071	−0.604
Evaluation	9	16	12.29 ± 1.6	−0.088	−0.638
Ethics	9	16	12.61 ± 1.4	0.167	−0.507

*Note:* The distribution of attitude scores was approximately symmetric (skewness ≈ 0) and platykurtic (Kurtosis < 0), indicating a concentration of scores around the mean with lighter tails than a normal distribution.

Abbreviations: AI = artificial intelligence; SD, standard deviation.

### 3.3. Common Method Bias Test Results

The results of Harman’s single‐factor test analysis showed that there were 12 factors with eigenvalues greater than 1, and the first factor explained 29.2% of the variance for Prosecco soilures subsp. soilures, which is below the critical value of 40%, indicating no severe common method bias phenomenon.

### 3.4. Correlation Analysis of Clinical Nurses’ Change Fatigue, AI Literacy, and AI Application Attitudes

The results in Table 3 show that change fatigue was moderately to strongly correlated with all dimensions of AI literacy (*r* ranging from −0.479 to −0.600, all *p* < 0.01) and with the overall AI application attitude (*r* = −0.558, *p* < 0.01). These negative correlations suggest that nurses who reported higher levels of change fatigue consistently reported lower AI literacy and less positive attitudes toward AI. Conversely, AI literacy was moderately positively correlated with AI application attitude (*r* = 0.571, *p* < 0.01), indicating that greater AI competency is associated with more favorable attitudes. The strong correlations among the four AILS subscales (*r* between 0.539 and 0.843) support the internal coherence of the AI literacy construct.

**TABLE 3:** Correlation analysis of the clinical nurse change fatigue scale, AILS scale, and AI application attitude scale.

Project	Change fatigue	ASUAITIN	Consciousness	Using	Evaluation	Ethics	AILS
Change fatigue	1	−0.558^∗∗^	−0.523^∗∗^	−0.500^∗∗^	−0.525^∗∗^	−0.479^∗∗^	−0.600^∗∗^
ASUAITIN		1	0.466^∗∗^	0.531^∗∗^	0.476^∗∗^	0.474^∗∗^	0.571^∗∗^
Consciousness			1	0.588^∗∗^	0.539^∗∗^	0.553^∗∗^	0.830^∗∗^
Using				1	0.652^∗∗^	0.700^∗∗^	0.843^∗∗^
Evaluation					1	0.649^∗∗^	0.832^∗∗^
Ethics						1	0.835^∗∗^
AILS							1

*Note:* Significant correlation at the 0.01 level (two‐tailed).

### 3.5. Analysis of the Mediating Effect of Clinical Nurses′ AI Literacy Between Change Fatigue and AI Application Attitude

**FIGURE 1:**
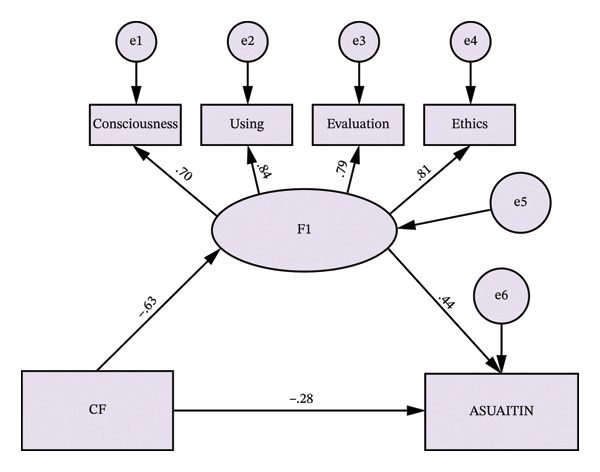
The mediating effect mode (standardized model). Note: AILS, Artificial Intelligence Literacy Scale; ASUAITIN, Attitude Scale Towards the Use of Artificial Intelligence Technologies in Nursing; CF, change fatigue. All paths are significant.

A SEM was established with change fatigue as the independent variable, AI literacy as the mediating variable, and AI application attitude as the dependent variable, as shown in Figure 1. The model fit indices were: *χ* ^2^/df = 2.761 < 3, RMSEA = 0.062, NFI = 0.983, CFI = 0.989, and IFI = 0.989, all within acceptable ranges, indicating good model fit. The standardized path coefficients indicated that change fatigue had a significant negative direct effect on AI application attitude (*β* = −0.280, *p* < 0.001). Change fatigue also negatively influenced AI literacy (*β* = −0.631, *p* < 0.001), which in turn positively affected AI application attitude (*β* = 0.440, *p* < 0.001). Bootstrap analysis revealed a significant indirect effect (*β* = −0.278, 95% CI: [−0.367, −0.199]). The total effect of change fatigue on attitude was *β* = −0.558. The indirect effect accounted for 49.8% of the total effect, confirming AI literacy’s significant partial mediating role.

The indirect effect of −0.185 indicates that, for every one‐unit increase in change fatigue, AI application attitude decreases by 0.185 units through its negative effect on AI literacy. The fact that the indirect effect (37.7% of total) and direct effect (62.3%) are both significant indicates a partial mediation, suggesting that change fatigue influences AI attitudes both directly and indirectly by reducing AI literacy.

### 3.6. Robustness Check for Common Method Bias

To further assess the potential impact of common method bias, the unmeasured latent method factor approach was applied. A method factor loading on all observed indicators was added to the original mediation model. The model fit of this constrained model did not show meaningful improvement compared to the original model (ΔCFI = 0.007, ΔTLI = 0.006). More importantly, the significance, direction, and magnitude of all hypothesized structural paths remained virtually unchanged. These results suggest that common method bias is unlikely to be a substantial threat to the interpretation of the primary findings.

## 4. Discussion

### 4.1. Current Status of Change Fatigue, AI Literacy, and Attitudes Toward AI Application Among County‐Level Nurses

The study reveals that clinical nurses in county hospitals exhibit moderate‐to‐high levels of transformation fatigue (29.27 ± 6.98 points, 69.7% score rate), surpassing the average transformation fatigue observed among nurses in tertiary hospitals [22]. In recent years, county hospitals have undergone multiple transformations including healthcare reforms, technological advancements, and management model shifts, all of which continuously impact nurses’ work patterns and psychological well‐being. Moreover, the staffing of nursing personnel in county hospitals is often less adequate compared to tertiary hospitals, forcing nurses to shoulder heavier responsibilities. In this study, 67.61% (311 nurses) required working over four night shifts monthly, maintaining prolonged high‐intensity workloads that contribute to physical and mental exhaustion. Additionally, 55% (253 nurses) held junior or lower professional titles, indicating limited career development opportunities in county hospitals. This professional uncertainty may lead to career uncertainty, further exacerbating transformation fatigue. Furthermore, the lack of effective psychological support mechanisms in county hospitals means nurses’ work‐related stress and negative emotions often go unaddressed, with accumulated pressures ultimately intensifying transformation fatigue.

The overall AI literacy score (49.82 ± 5.27 points, 59.31% participation rate) at county‐level hospitals remains at a moderate level, lower than findings from concurrent studies in tertiary hospitals [23]. This gap may stem from resource allocation disparities and limited access to advanced technologies in county healthcare facilities. Compared to top‐tier hospitals, county hospitals often lack adequate AI‐equipped devices and technical support, which restricts nurses’ understanding and application of AI technologies. Notably, only 58 nurses (12.61%) in county hospitals received AI training, highlighting the relative scarcity of professional development resources and learning opportunities in primary care settings, thereby hindering effective AI competency enhancement [8]. Furthermore, the work environment and task priorities in county hospitals may dampen nurses’ enthusiasm for AI‐related learning, resulting in insufficient investment in AI literacy development. These disparities underscore the critical need for specialized training programs and support measures for county hospital nurses to bridge the gap with high‐level medical institutions.

The overall AI application attitude score (45.26 ± 2.45 points, 60.24% score rate) remains at a moderate level, lower than that of nurses in tertiary hospitals. Notably, the “negative attitude” subscale of the ASUAITIN captures nurses’ concerns and anxieties about AI, including fears of job replacement, ethical risks, loss of professional autonomy, and increased technological burden. In our sample, although the absolute mean score of the positive attitude subscale (23.07 ± 5.54) was slightly higher than that of the negative attitude subscale (22.19 ± 4.04), the scoring rate for the negative attitude subscale (73.97%) was substantially higher than that for the positive attitude subscale (51.27%). This indicates that nurses in county‐level hospitals, while rationally recognizing the potential benefits of AI, are emotionally more driven by apprehension than by optimism. Such attitudes may stem from nurses’ limited understanding of AI technology, concerns about potential risks, and reliance on traditional work patterns. Additionally, county hospitals may face challenges in promoting AI implementation, including insufficient publicity efforts and limited practical application scenarios, which diminish nurses’ intuitive perception of AI’s value. The high negative attitude score has important practical implications: it suggests that standalone AI literacy training may be insufficient to shift attitudes. Interventions must also directly address emotional concerns through transparent communication about AI’s role (e.g., AI as a decision support tool rather than a replacement for nurses), involvement of frontline nurses in AI implementation decisions, and clear organizational guarantees regarding job security and professional autonomy. Therefore, while enhancing county nurses’ AI literacy, it is essential to boost their confidence through real‐world case demonstrations and hands‐on technical experience activities, thereby gradually improving their attitude toward AI adoption.

### 4.2. Core Summary of Findings

This study tested a theoretical model grounded in the COR theory within a specific and underexplored context: county‐level hospitals in China. The primary findings confirm a significant serial relationship. Higher levels of change fatigue among nurses are associated with lower AI literacy, which in turn is linked to less positive attitudes toward AI application. AI literacy was found to be a significant mediator, accounting for a substantial portion (37.7%) of the total relationship between change fatigue and AI attitudes. This illuminates a potential cognitive pathway—resource depletion impairing competency development—through which the cumulative burden of organizational change may shape receptivity to technological innovation.

### 4.3. Dialog With and Comparison to Existing Evidence

#### 4.3.1. Change Fatigue and Technology Attitudes: Support and Contextual Extension

Our finding that change fatigue shows a strong negative association with AI application attitudes aligns with broader organizational literature on change resistance and innovation fatigue [11, 24]. However, it is important to note that some studies have reported nonlinear effects of organizational change on technology acceptance. For instance, a moderate level of change pressure may occasionally enhance adaptive performance by prompting individuals to develop coping strategies [25–28]. Nevertheless, our results suggest that in the context of county‐level hospitals—where changes are frequent, simultaneous, and resource‐intensive—the cumulative effect is predominantly negative, likely exceeding any theoretical “optimal” level of change pressure. This finding challenges the assumption that change fatigue operates uniformly across settings and underscores the need for context‐specific thresholds in future research.

#### 4.3.2. The Mediating Role of AI Literacy: Validation and Pathway Specification

The confirmed mediating role of AI literacy substantiates core tenets of technology acceptance models (TAM), which emphasize perceived ease of use and usefulness [29]. However, our findings extend and refine TAM by demonstrating that these perceptions are not formed in isolation; they are contingent upon nurses’ cognitive resource availability, which is eroded by change fatigue. This suggests that existing TAM‐based interventions that focus solely on highlighting AI’s usefulness may be insufficient. A critical integration of the COR theory with TAM is warranted, as resource depletion can act as a boundary condition limiting the effectiveness of traditional technology acceptance strategies.

### 4.4. Divergences and Contextual Explanations

Notably, the mean scores for both AI literacy and AI application attitudes in our sample were lower than those reported in studies from tertiary hospitals [23]. This discrepancy, while consistent with resource gradient explanations, also invites comparison with international studies that have reported unexpectedly high AI acceptance among nurses in other low‐resource settings. For example, a recent study in rural Egypt found relatively positive AI attitudes despite similar resource constraints [16]. One potential explanation for this divergence is the difference in policy support and top‐down messaging: Egyptian nurses may have received stronger government‐level endorsements of AI, whereas Chinese county‐level nurses face more ambiguous messaging amid rapid but fragmented reforms. This comparison highlights that resource constraints alone do not determine attitudes; organizational communication and change management strategies are critical moderators, a point we will return to in our practical recommendations.

### 4.5. Implications for Multilevel Practice

#### 4.5.1. For Nursing Education and Professional Development

Current AI training for nurses often focuses solely on technical skills. Our mediation model (indirect effect: −0.185, 37.7% of total effect) suggests that without addressing change fatigue, AI literacy training may have limited impact. We recommend:

(a) Integrating resilience‐building modules into pre‐licensure curricula (2 h, over 4 weeks) using case‐based scenarios to teach psychological resource management [30, 31].
(b) Offering just‐in‐time coping workshops for nurses with high change fatigue (CFS score > 35), delivered monthly by trained peer supporters [21, 32].
(c) Embedding AI literacy into existing continuing education via 10‐min AI examples at monthly grand rounds, reducing cognitive load through familiar clinical contexts [33, 34].

#### 4.5.2. For Hospital Management and Organizational Development

Based on our findings (12.61% AI training rate; 73.97% negative attitude scoring rate), we propose five evidence‐based strategies:

(a) Change fatigue monitoring. Administer the 6‐item Change Fatigue Scale quarterly; target reducing mean scores from 29.3 to ≤ 25.0 in 12 months. Longitudinal evidence shows shift‐work fatigue is measurable and modifiable [35].
(b) Paced change protocols. Conduct change impact assessments with frontline nurses before AI introduction; stagger major changes by ≥ 3 months to avoid “change avalanche.” Implementation science supports staggered timelines to reduce staff resistance [36].
(c) Codesign with nurses. Form a nurse‐led AI advisory committee (6–8 members, rotating every 6 months). Participatory design empowers frontline staff; nurse‐led AI governance has succeeded at leading health systems [37].
(d) Low‐burden AI literacy training. Replace all‐day workshops with microlearning modules (10–15 min, via WeChat/app). Cover four topics over 8 weeks. Microlearning AI education yields significant knowledge gains (Cohen’s *d* = 0.65) and high satisfaction among nurses [34, 38].
(e) Directly address emotional concerns. Issue a leadership‐signed AI transparency charter (e.g., “AI assists, not replaces, clinical decisions”). Transparent communication reduces technostress and bridges clinician trust gaps [39].

Implementation timeline: Months 1–2: monitoring + advisory committee; Months 3–4: microlearning modules; Months 5–6: paced change + charter; Month 12: reassess.

#### 4.5.3. For Ethical and System‐Level Implementation

Ethical Dimension: Our findings reinforce the principles of responsible innovation. An ethical approach to AI integration must prioritize nurse well‐being and fair process, not solely efficiency gains [40]. Proactively managing change fatigue and building literacy is an ethical imperative to ensure just and sustainable implementation.

System‐Level Cost‐Benefit Consideration: From a health systems perspective, the model implies that upfront investments in interventions to reduce change fatigue and build AI literacy are not merely supportive but potentially cost‐effective. These investments may prevent the high costs associated with implementation failure, low adoption rates, and workforce burnout, thereby improving the long‐term return on investment for AI technologies [13].

## 5. Limitations

This study has several limitations that should be acknowledged. The cross‐sectional design precludes causal inference; reverse relationships (e.g., higher AI literacy reducing perceived change fatigue) are possible, and future longitudinal or experimental studies are needed to establish temporal direction.

Data were collected from a single county‐level hospital in Eastern China using convenience sampling. This specific setting (e.g., recent hospital relocation, relatively affluent region) limits generalizability to other county hospitals in Central or Western China, or to primary care settings in other countries. The unique cultural and organizational context of Chinese healthcare, characterized by hierarchical decision‐making and rapid policy‐driven reforms, may also shape change fatigue and AI attitudes differently than in other cultural settings. Caution is therefore needed when extrapolating findings. Most participants had minimal hands‐on AI exposure (only 12.61% had received AI training), so measured attitudes reflect preadoption perceptions rather than postuse evaluations. Future research should replicate the model in settings with direct clinical AI experience.

Despite controlling for key demographics, unmeasured confounders (e.g., personality traits, organizational support) may exist. Restricted variance in attitude scores (platykurtic distribution) may have attenuated effect estimates. Common method bias cannot be fully ruled out given the self‐report design, although statistical tests suggested it was not severe.

Finally, as a purely quantitative study, we lack qualitative data that could explain why negative attitude scores (73.97% scoring rate) substantially outweigh positive ones (51.27%). Future mixed‐methods research using focus groups or interviews could explore nurses’ lived experiences and specific concerns about change fatigue and AI.

## 6. Conclusion

This study makes several unique theoretical and practical contributions.

Theoretically, it extends the COR theory to AI acceptance in nursing. It empirically demonstrates a novel pathway. Generalized organizational change fatigue indirectly impairs attitudes toward AI. It does so through the erosion of domain‐specific AI literacy. This bridges two previously siloed research areas. One is organizational psychology focused on change and stress. The other is health informatics focused on technology acceptance. This offers an integrated explanatory framework. Unlike prior studies that focused on individual‐level factors such as age, education, or prior technology experience, our model reveals that the organizational context is a critical antecedent. Specifically, cumulative change fatigue conditions the effectiveness of AI literacy interventions.

Practically, the findings challenge the assumption that standalone AI skills training is sufficient. In resource‐constrained county‐level hospitals, only 12.61% of nurses had received any AI training. Negative attitude scoring rates reached 73.97%, substantially outweighing positive ones at 51.27%. A dual intervention strategy is required. First, implement paced change management and psychological support to mitigate resource depletion from change fatigue. Second, deliver low‐burden, stratified AI literacy programs that also address emotional concerns such as job replacement fears and ethical risks. These strategies are not merely supportive but potentially cost‐effective. Upfront investments may prevent the high costs of implementation failure and workforce burnout. By balancing resource preservation and resource building, nursing administrators can foster more positive and sustainable attitudes toward AI technology in primary care settings.

## Author Contributions

Ming Yu and Lingling Jiang conceived and designed the study. Ming Yu, Rong Yu, Mengjia Zhou, and Xiaoli Fan performed data collection and management. Ming Yu and Jing Ji conducted the statistical analysis. Ming Yu drafted the initial manuscript. Lingling Jiang (corresponding author), Ronghui Geng, Suping Cai, Lili Jiang, and Rong Yu critically revised the manuscript for important intellectual content. Ming Yu and Rong Yu are co‐authors.

## Funding

This work received no funding.

## Disclosure

All authors read and approved the final version of the manuscript.

## Ethics Statement

This study was approved by the Ethics Committee of Rudong Hospital (No. 2022001). All methods were performed in accordance with the relevant guidelines and regulations, including the Declaration of Helsinki.

## Consent

Informed consent was obtained from all participants after they were informed about the study purpose and data confidentiality.

## Conflicts of Interest

The authors declare no conflicts of interest.

## Data Availability

The datasets generated and analyzed during this study are available from the corresponding author upon reasonable request.
